# Oculist or Optician?

**Published:** 1896-10

**Authors:** 


### OCULIST OR OPTICIAN?

The *Journal of Medicine and Science* for August contains a good article by Dr. C. E. Norton, of Lewiston, Maine, upon the "Necessity of Having the Eyes Examined for Glasses by an Oculist." The writer very clearly points out that the optician is not the person to prescribe glasses. He divides present-day opticians into two classes: those "whose profession is the manufacture of glasses, which they make for patients only on the prescription from an oculist," and those "who make glasses and examine eyes, and pretend to fit glasses to them." It is this latter class which the patient should studiously avoid. Glasses are used to improve vision, either near or distant, or both; also, to preserve vision, and to relieve a variety of reflex functional diseases, particularly headaches. It is not necessary to enumerate the many causes of bad vision, which glasses, properly selected, are used to correct, and a knowledge of which the optician is not expected to possess and cannot possess. Because an optician makes and handles all kinds of spectacles and lenses, he is no more capable of prescribing glasses to correct refractive errors than a blacksmith is a proper person to perform a surgical operation.

"Or again, as a druggist keeps all kinds of medicine and compounds them with even greater skill than the average physician, it might be argued that the druggist, instead of the doctor, should prescribe medicines. But the fallacy of this argument every physician can see. The prescription of glasses is no more mechanical than the prescription of medicines. A physician may find it necessary to administer a cathartic, but it requires some skill on his part to know which cathartic best meets the indications in the case before him. In the same way, after the oculist has found and measured the optical error, it requires skill to know exactly what glasses should be prescribed in the treatment of the case."

The author impresses upon general physicians the duty of referring patients to a competent oculist, instead of the optician. His paper is a strong argument, and the several cases which he describes, illustrating the abuse of the optician's art, materially help to sustain his points.

